# Outreach and online training services at the *Saccharomyces* Genome Database

**DOI:** 10.1093/database/bax002

**Published:** 2017-02-26

**Authors:** Kevin A. MacPherson, Barry Starr, Edith D. Wong, Kyla S. Dalusag, Sage T. Hellerstedt, Olivia W. Lang, Robert S. Nash, Marek S. Skrzypek, Stacia R. Engel, J. Michael Cherry

**Affiliations:** Department of Genetics, Stanford University, Stanford, CA 94305, USA

## Abstract

The *Saccharomyces* Genome Database (SGD; www.yeastgenome.org), the primary genetics and genomics resource for the budding yeast *S. cerevisiae*, provides free public access to expertly curated information about the yeast genome and its gene products. As the central hub for the yeast research community, SGD engages in a variety of social outreach efforts to inform our users about new developments, promote collaboration, increase public awareness of the importance of yeast to biomedical research, and facilitate scientific discovery. Here we describe these various outreach methods, from networking at scientific conferences to the use of online media such as blog posts and webinars, and include our perspectives on the benefits provided by outreach activities for model organism databases.

**Database URL:** http://www.yeastgenome.org

## Introduction

Model organism databases (MODs) are service organizations that curate published biological data on model organisms, making this data available to researchers, educators, and students. Model organisms such as yeast, fly and mouse are frequently used in biological research to produce data that reflects and predicts the biology of other organisms, such as humans (1, 2). To facilitate discovery and promote novel research within each research community, MODs assimilate diverse types of biological data from various sources, organize them into a comprehensible format, and make them easily accessible to users online, enabling researchers to sort through vast amounts of data and quickly locate key information. MODs are an essential resource for many scientists, and will only become more important as the pace of data production in biology continues to accelerate (3).

MODs play an important role in circulating information within communities and increasing awareness of research (4). A user who visits a single gene summary page at SGD, for example, can find information regarding the various functional, phenotypic, regulatory, interaction, and expression studies that have been performed on that gene and others (5–7). Moreover, by making clear citations for all curated data and providing community resources like searchable colleague directories and career postings, MODs increase awareness of what is being done, by whom in the community. This fosters communication between laboratories and helps avoid redundancy of effort. In essence, because of their roles in distributing published work and facilitating connections between scientists, MODs function as the central hub of their respective model organism research communities.

SGD, as the center of the yeast research community, has engaged in a variety of social outreach and communication efforts aimed at increasing awareness of the value of yeast in research, promoting growth and collaboration within our community, and improving user familiarity with our website. We connect with the community in diverse ways: direct contact with authors of yeast research, participating in conferences and hosting workshops, active involvement in social media, and production of online help documents, video tutorials, educational wiki pages, webinars and blog posts. Here, we discuss these outreach efforts and the methods behind them. Additionally, we provide our perspectives on the overall effort vs. value added for MOD outreach in general.

## SGD blog and social media

### SGD blog

**Figure 1:**
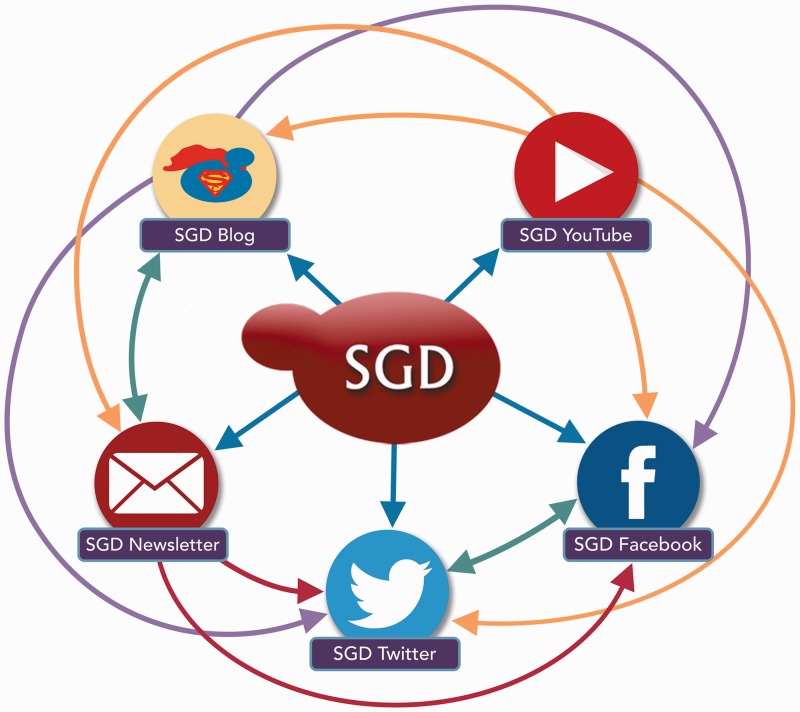
The SGD uses a variety of outreach and social media platforms to disseminate news and information to our user community. To increase readership and reach the broadest possible audience, content posted on one outreach platform is often publicized, announced or re-posted on other outreach platforms. Colored arrows indicate the directions in which content is pushed from one platform to another.

The SGD blog (www.yeastgenome.org/blog) is our primary method of informing the SGD user community about current news and recent developments. Whenever SGD releases a significant update, such as new tools, new data, a change of website layout, etc., we inform SGD users via blog posts on the SGD homepage, which are then disseminated via Facebook and Twitter. We also use these platforms to distribute our community newsletter, which summarizes SGD’s latest activities (Figure 1). In addition to website updates and newsletters, blog posts inform our users about SGD workshops, webinars, new help videos, general announcements, and more. SGD team members at all levels contribute to updating the blog with new posts and information.

### Research spotlight

**Figure 2:**
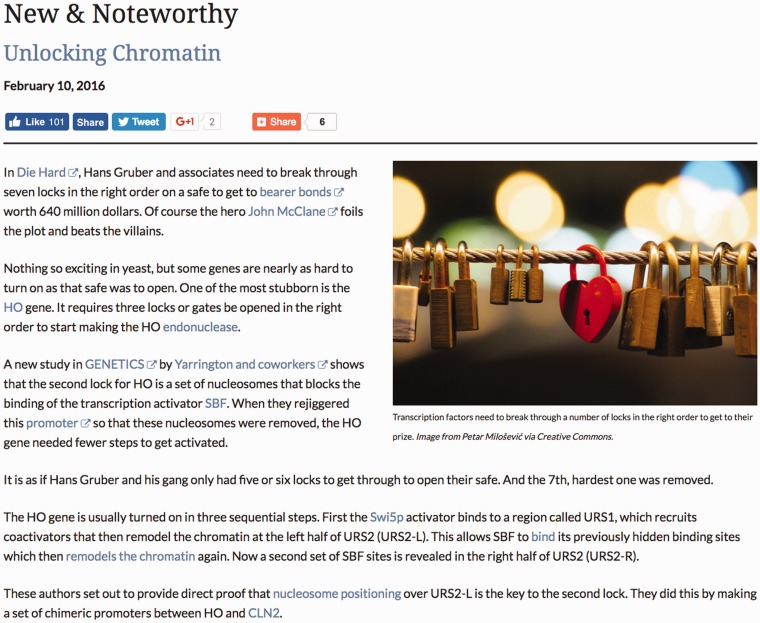
A key feature of the SGD blog is the Research Spotlight, which use real-world analogies to highlight recent yeast research articles in an informal and engaging manner.

A key feature of the SGD blog is the Research Spotlight (www.yeastgenome.org/category/research-spotlight; Figure 2). These posts highlight recent yeast research articles in an informal and engaging manner, using real-world analogies to explain article content in ways that can be enjoyed by researchers and students, as well as citizen scientists. Research Spotlights place particular emphasis on publications that demonstrate new findings in basic science, or the relevance of yeast to biomedical research. We post new Research Spotlights an average of three to four times per month, with 184 posted over the past 4 years. Each Research Spotlight is planned, composed, proofread, and published on the SGD blog from the joint efforts of three team members, who collectively spend no more than 6 work hours per Spotlight. Overall, feedback from the community has been positive: many authors communicate their enthusiasm to SGD about having their work highlighted, and our Research Spotlight announcements are frequently shared or ‘liked’ by the public on social media.

### Social media

SGD is active on major social media platforms (Table 1). Among these, the SGD Facebook page and Twitter feed serve as important communication outlets. We use these platforms to distribute news items featured on the SGD homepage such as Research Spotlight blog posts, new help videos on the SGD YouTube channel, news about Nobel prizes awarded to yeast researchers, newly published articles and significant upcoming conferences. We also employ social media to advocate for yeast research, facilitate interaction within the yeast community, and engage users regarding scientific topics in general.

**Table 1:** A variety of social media platforms are used by the SGD

Platform	URL
SGD Facebook	http://facebook.com/yeastgenome
SGD Twitter	https://twitter.com/yeastgenome
SGD YouTube	https://www.youtube.com/Saccharomyces GenomeDatabase
SGD LinkedIn	https://www.linkedin.com/company/ saccharomyces-genome-database
SGD Google+	https://plus.google.com/+Saccharomyces GenomeDatabase

On average, we update the SGD Twitter feed eight times per week and the SGD Facebook page four times per week. New posts on these platforms are generally written by one of two designated SGD team members, who spend collectively 1–2 h/week maintaining our social media presence. In our experience, Twitter elicits more written feedback from the community than Facebook. The comments we receive on Twitter are often about SGD’s tools or content, lighthearted anecdotes about interesting uses of yeast, recent trends in model organism research, or photos and summaries of a user’s experience at a SGD workshop or seminar. We currently have > 1500 ‘Likes’ on Facebook, and the same amount followers on Twitter, each representing about one-eighth of the users registered as ‘Colleagues’ at SGD. Although our Facebook and Twitter audiences are roughly the same size, they do represent different demographic groups. Facebook is equally split between male and female, with approximately one-half of the users between the ages of 25 and 34. Twitter, however, is skewed toward a younger male audience: male followers outnumber females two-to-one, and two-thirds are between the ages of 25 and 34.

## Online tutorials, webinars and YouTube

**Figure 3:**
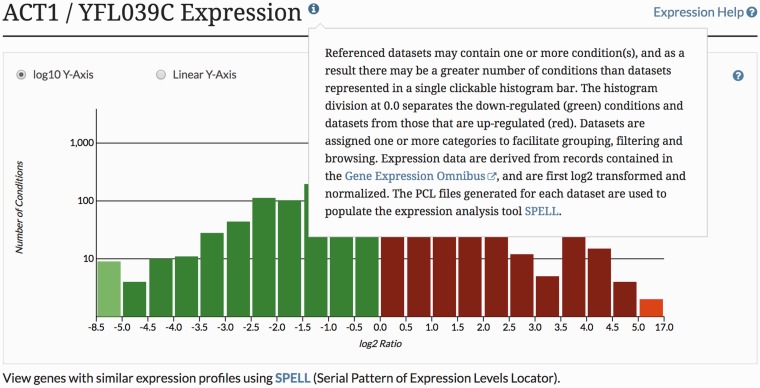
The SGD provides context-specific help throughout the SGD website, enabling quick *in situ* access to explanations of various tools and features.

MODs offer a variety of tools and resources that facilitate data analysis and research (1). Many of these tools can go unnoticed or underutilized, as busy users often forego learning to use tools that would otherwise benefit their work. It is therefore of paramount importance to increase awareness of available tools and provide timely access to quick, easy-to-follow tutorials so as to encourage their use. To increase familiarity with tools and resources, SGD provides extensive written tutorials (www.yeastgenome.org/help). In recognition of the need for convenience and simplicity, SGD also provides context-specific help throughout the SGD website, enabling quick *in situ* access to explanations of various features (Figure 3).

### Video tutorials

**Figure 4:**
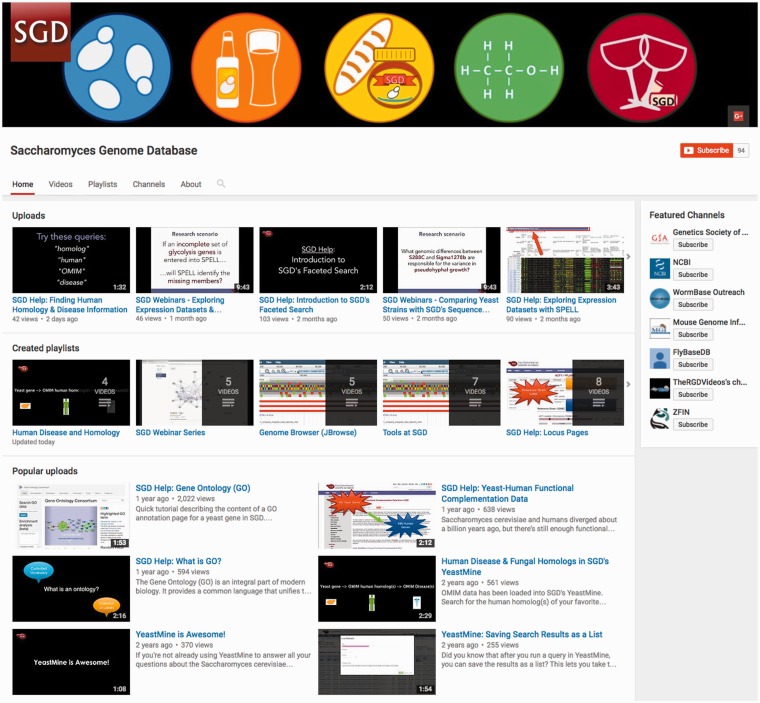
The SGD distributes video tutorials via the SGD YouTube channel (youtube.com/SaccharomycesGenomeDatabase).

Although online help pages are sufficient for many, SGD has a broad user base that includes new students, experienced educators, and seasoned researchers. For this reason, we provide diverse help resources that accommodate a variety of learning levels and styles. To maximize user familiarity with our tools, we distribute video tutorials via our YouTube channel (Figure 4; youtube.com/SaccharomycesGenomeDatabase). Videos are a particularly useful medium in which to create tutorials as they can illustrate what the natural workflow for a tool should look like, the sequence of events leading up to executing a tool’s specific function, and precisely where on a page users should be clicking in order to navigate the tool’s interface. SGD prioritizes help videos that cover newly released tools and/or address common questions (FAQs) received from our user community. We will then use these videos to respond to users’ questions and also make the videos available on our YouTube channel for general consumption. In this way, video tutorials serve a dual function: they facilitate the use of our tools and resources, and serve as an avenue through which we actively engage our user community.

**Figure 5:**
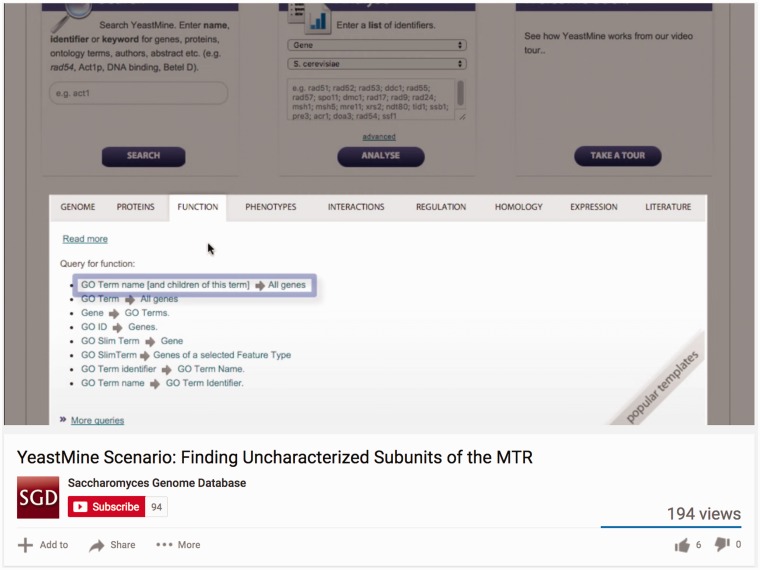
Camtasia software (www.techsmith.com/camtasia.html) has been used by the SGD to add simple effects and annotations to video tutorials in order to enhance clarity of the presented material.

In expanding the SGD YouTube channel, we have found that professional video editing software is not necessary for making high-quality help videos. To date, SGD has employed Camtasia (www.techsmith.com/camtasia) as a cost-effective platform to create video tutorials, although any video editing software capable of the following tasks would be sufficient: (i) recording activities on a computer screen, (ii) editing recorded audio files to align with video recordings and (iii) adding simple effects and annotations such as transition fades, arrow pointers, image/figure insertions and text boxes. Camtasia has also been useful for creating animated figures that add clarity (Figure 5). An inexpensive handheld microphone is an effective way to add clear narration to videos. In general, a 2–3-min video can take 3–5 h to create, depending on the topic, the need for figures and animations, and the amount of time spent scripting and recording audio. The number of annotations needed to make a clear video tutorial is also an important factor—a simple video with narration takes less time to make than a highly annotated video with arrow pointers, circles, zooms, and transitions. As such, making tutorials for tools with easily navigated interfaces and straightforward functions requires minimal annotations. In contrast, videos for tools with complicated interfaces or diverse functions require extensive annotations to be clear enough to follow, greatly increasing production time.

### SGD webinar series

Webinars, like help videos, are flexible media for MODs to connect with their user communities, disseminate information, and help users become familiar with MOD resources. In 2016, we initiated the SGD Webinar Series: a growing set of interactive online seminars aimed at demonstrating aspects of the database and the value of yeast as a model organism. SGD webinars typically focus on a single tool, such as YeastMine (8; yeastmine.yeastgenome.org) or JBrowse (9; yeastgenome.org/browse), but have also focused on topics such as the importance of SGD to research and the purpose of biocuration. We have found that short timeframes, 15 min or less, have been sufficient to both deliver quality step-by-step tutorials and accommodate the tight schedules of busy viewers. This longer format, relative to our short 1–3-min help videos, enables coverage of SGD tools in greater depth. Further, we often integrate practical research scenarios or real-life user questions into each webinar in order to more fully engage the audience.

Hosting webinars requires a video conferencing or webinar service, which provides the platform through which content is delivered and also offers secondary features important to the overall webinar experience. Because these services vary in cost and provided features, selecting a service with the right complement of features is essential. We recommend any webinar service that has an appropriate maximum number of attendees, recording options, question-and-answer (Q&A) platform and clear video/audio quality. Other features, such as mobile access to the webinar or a streamlined registration process, may warrant consideration depending on the estimated number of attendees. SGD currently utilizes BlueJeans Primetime (bluejeans.com/primetime) to host webinars. For webinar registration, we use Google Forms then send confirmation emails to registrants manually.

To create the actual webinar content, SGD employs PowerPoint (www.microsoft.com) with embedded prerecorded videos made using Camtasia. The video files are split into individual steps and incorporated into the presentation. SGD staff members narrate the video live. Using video files embedded into PowerPoint requires more time for recording and editing, but provides three important benefits: (i) it allows the webinars to feature video annotations, such as arrows, zooms and highlights, which add clarity, (ii) it ensures that the audience is able to resolve all the features of the tool being demonstrated, which is especially important for viewers that receive poor video quality and (iii) it safeguards against technical hurdles, such as server downtimes or interruptions, when showcasing the SGD website or a specific tool. A summary of the resources used to produce our webinars is provided in Table 2.

**Table 2:** Various external resources are used to produce webinars for the SGD Webinar Series

Resource	Purpose	URL
BlueJeans Primetime	Webinar platform	https://bluejeans.com/primetime
Microsoft PowerPoint	Presentation software	https://products.office.com/en-us/powerpoint
Camtasia	Pre-recording content	https://www.techsmith.com/camtasia.html
Google Forms	Attendee registration	https://www.google.com/forms

To advertise for our webinars, we advertise on our homepage blog, send email announcements to our opt-in contact list, and post notices on social media. A single presenting speaker, with a supporting team of moderators, hosts each webinar. In addition to sharing many of the same benefits as help videos, webinars are distinguished by the fact that they are interactive. We encourage audience members to ask questions throughout the event, which are answered by the supporting moderators. To accommodate an international user community and enable attendance from multiple time zones, we conduct webinars in the morning hours of US Pacific time. Webinars are then posted on the SGD YouTube channel, so that those who cannot attend the live webinar can view it later at their convenience.

Each webinar takes ∼12–15 work hours to produce. Webinars require more time than help videos because of the need for rehearsal, advertising, attendee registration etc., but these factors have not been prohibitive to hosting webinars on a monthly-to-bimonthly schedule. As with help videos, the time investment required for each webinar decreases as staff becomes more proficient at producing them. Webinars have overall served as an inexpensive platform for SGD to deliver tutorials, connect with our user community, and promote yeast research. Adding to this, completed webinars are recorded and uploaded to the SGD YouTube channel where they continue to benefit hundreds of users long after the event. These outcomes are highly valuable to SGD and suggest the value of webinars for other MODs in general.

## Meetings and conferences

Scientific conferences and other meetings within the research community are key settings for MODs to engage in outreach activities. SGD participates in various conferences by hosting workshops and exhibits to teach users about our database and inform them about ongoing projects. These workshops and exhibits have become familiar gathering points for existing users, and also attract new users. Workshop topics include the availability of new data types, upcoming features and website changes, curation projects and strategies, practical use-cases, and live tool demonstrations. The importance of these workshops stems not only from their content, but also from the resulting interaction SGD has with the user community. Where users may be excited about an upcoming feature, concerned about an incoming website change, or confused about one of our online tools, workshops enable users to communicate their thoughts on our ongoing projects. Thus, workshops facilitate SGD’s outreach efforts by giving users an inside look at the work being done by SGD, and establishing a forum for SGD to address questions and comments from the community.

### Exhibit booths

In addition to workshops, we host exhibit booths or tables at conferences to provide one-on-one assistance with SGD-related questions and promote use of the database. Here, SGD staff deliver personalized on-the-fly tutorials regarding features of the website and direct visitors to resources relevant to their specific projects. To draw more visitors to the booth while spreading information about SGD, we provide informational flyers about our website along with promotional handouts, such as MOD-themed stickers or bottle openers etched with the SGD logo and URL. Additionally, to increase booth participation, we sometimes challenge visitors to complete a fun task, such as a science-themed crossword puzzle, in order to earn one of our highly coveted SGD T-shirts or hats. Because much of the crossword puzzle (or other exercise) can be completed by going through the SGD website, this outreach strategy—which has been completed over the years by hundreds of participants—has been particularly successful at encouraging visitors to explore the website. By drawing more visitors to our booths and tables, these promotional elements provide additional opportunities to discuss our database with individual researchers and create more long-term users for SGD.

## Direct author contact

Two-way communication between MODs and scientists is key to efficient biocuration (10, 11). To prioritize significant discoveries made within the yeast community, SGD contacts authors of recent *S. cerevisiae*-related publications to inquire about their novel characterizations and data. We employ a semi-automated system that sends emails to corresponding authors every 2 weeks. In these emails, we ask authors to tell us about the content of their articles using a simple web submission form (www.yeastgenome.org/cgi-bin/submitData.pl), which includes straightforward questions such as:

- Does this article contain novel characterizations of the function, role or localization of a gene product(s)?
- If this article focuses on specific genes/proteins, please identify them.
- Does this study include large-scale datasets that you'd like to see incorporated into SGD?
- Is there anything else that you would like us to know about this article?

We call this our ‘Fast Track’ system, and have modeled it after the ‘Fast-Track Your Paper’ tool at FlyBase (12; flybase.org/submission/publication/). Participation is consistently high: ∼60% of the authors we contact respond to our request. Many are eager to have their data represented by SGD and help us remain up-to-date. Overall, this system has expedited our curation process by helping to prioritize articles and by highlighting specific data within the literature.

## Collaboration with *Genetics Society of America* journals

The need to look up unfamiliar gene names while reading journal articles can be a significant time burden to researchers. To accelerate learning and connect researchers to relevant data, SGD annotates online journal articles published in the *Genetics Society of America* journals GENETICS and G3: Genes | Genomes | Genetics. SGD biocurators associate any yeast genes listed in these articles to their respective locus summary page at SGD, providing better context and reducing the time needed by readers to look up unfamiliar terms. To provide these annotations, we work through a semi-automated markup pipeline in which an automated script searches for yeast genes within pre-production articles and associates them with an initial hyperlink. SGD biocurators then review these hyperlinks, adding and modifying annotations where appropriate, and later perform a second review when the final PDF version of the article has been prepared. Using this process, we annotate an average of 90–100 articles per year.

## Concluding remarks

SGD has engaged in a variety of outreach efforts to improve user familiarity with our website, promote growth within the yeast community, and increase awareness of the value of yeast to biomedical research. We find these efforts to be feasible and sustainable ways for MODs to provide outreach, and encourage MODs to adopt similar strategies. Moving forward, we will continue to develop our outreach services to further promote the use of SGD among students, educators and the research community as a whole.

## Funding

National Human Genome Research Institute at the United States National Institutes of Health (U41 HG001315). The content is solely the responsibility of the authors and does not necessarily represent the official views of the National Human Genome Research Institute or the National Institutes of Health. The funders had no role in design, data processing, implementation, decision to publish or preparation of the article.

*Conflict of interest*. None declared.
